# Supplementary material for: Development of core outcome sets for vision screening and assessment in stroke: a Delphi and consensus study
Source: BMJ Open. 2019 Sep 8;9(9):e029578. doi: 10.1136/bmjopen-2019-029578 (PMC6738691; doi:10.1136/bmjopen-2019-029578)
Supplement: Supplementary data [file bmjopen-2019-029578supp001.pdf]

Supplementary Table 1 General domains for screening

Domains	Delphi result		Consensus vote
	1-3 %	7-9 %	In / Out
Case history – asking patient an open question, e.g. Do you have any problem with your vision and, if yes, what is this?	0	98	In
Case history – asking patient very specific questions about the different visual problems that can occur	0	98	In
Case history – asking carer open questions, e.g. In your opinion, does the individual have any problem with their vision and, if yes, what is this?	0	90.2	In
Case history – asking carer specific questions about the different visual problems that can occur	0	92.1	In
Case history – ask about previous ocular history	0	94.1	In
Case history – ask about glasses	0	96.1	In
Observations – open comments in which the examiner lists anything they may notice	0	94.1	In
Observations – specific features prompting the examiner to look for particular issues	0	94.1	In
Letter charts or tests for visual acuity	2	78.4	In
Eye alignment position	3.9	94.1	In
Eye movement – ocular motility assessment	2	92.1	In
Command saccades	2	49	Out
Binocular vision assessment	7.9	43	Out
Eye alignment measurement	19.6	17.7	Out
Visual field assessment	0	98	In
Visual neglect assessment	0	92.1	In
Functional assessment, e.g. navigation, mobility	0	86.2	In
Reading assessment	3.9	56.9	In
Questionnaires for quality of life	24.5	17.6	Out
Pupil assessment	3.9	49	Out
Lid assessment	5.9	27.5	Out
Contrast sensitivity assessment	56.9	7.9	Out
Colour vision assessment	56.9	9.8	Out
Screen for visual memory and cognition	9.8	52.9	Out

Definition of consensus for including an item is defined as 70% of more of people scoring the item as 7-9 and fewer than 15% scoring it as 1-3.

Definition of consensus for excluding an item is defined as 70% or more of people scoring the item as 1-3 and fewer than 15% scoring it as 7-9.

All other score distributions indicate lack of agreement for inclusion of items.
