# Supplementary material for: Development of core outcome sets for vision screening and assessment in stroke: a Delphi and consensus study
Source: BMJ Open. 2019 Sep 8;9(9):e029578. doi: 10.1136/bmjopen-2019-029578 (PMC6738691; doi:10.1136/bmjopen-2019-029578)
Supplement: Supplementary data [file bmjopen-2019-029578supp002.pdf]

Supplementary Table 2 General domains for full assessment

Domains	Delphi result		Consensus vote
	1-3 %	7-9 %	In / Out
Case history – asking patient an open question, e.g. Do you have any problem with your vision and, if yes, what is this?	0	96	In
Case history – asking patient very specific questions about the different visual problems that can occur	0	98	In
Case history – asking carer open questions, e.g. In your opinion, does the individual have any problem with their vision and, if yes, what is this?	0	90.2	In
Case history – asking carer specific questions about the different visual problems that can occur	0	94.1	In
Case history – ask about previous ocular history	0	91.2	In
Case history – ask about glasses	0	94.1	In
Case history – ask about ability to make eye contact during conversations	11.8	43.2	Out
Observations – open comments in which the examiner lists anything they may notice	0	96	In
Observations – specific features prompting the examiner to look for particular issues	0	98	In
Letter charts or tests for visual acuity	0	94.1	In
Eye alignment position	0	94.1	In
Eye movement – ocular motility assessment	0	94.1	In
Voluntary and command saccades, Doll's head, convergence	0	84.3	In
Binocular vision assessment	0	90.2	In
Eye alignment measurement	0	74.5	In
Visual field assessment	0	98	In
Visual neglect assessment	0	94.1	In
Functional assessment, e.g. navigation, mobility	0	91.2	In
Reading assessment	0	88.2	In
Questionnaires for quality of life	11.8	27.5	In
Pupil assessment	0	84.3	In
Lid assessment	0	84.3	In
Contrast sensitivity assessment	19.6	23.5	Out
Colour vision assessment	15.6	27.5	Out
Visual perception	0	80.4	In
Fundus check	7.9	41.1	Out
Retinal photography/OCT	43.1	9.8	Out

Definition of consensus for including an item is defined as 70% of more of people scoring the item as 7-9 and fewer than 15% scoring it as 1-3.

Definition of consensus for excluding an item is defined as 70% or more of people scoring the item as 1-3 and fewer than 15% scoring it as 7-9.

All other score distributions indicate lack of agreement for inclusion of items.
